# Metabolite Profiling of Root Exudates of Common Bean under Phosphorus Deficiency

**DOI:** 10.3390/metabo4030599

**Published:** 2014-07-16

**Authors:** Keitaro Tawaraya, Ryota Horie, Saki Saito, Tadao Wagatsuma, Kazuki Saito, Akira Oikawa

**Affiliations:** 1 Faculty of Agriculture, Yamagata University, Tsuruoka 997-8555, Japan; E-Mails: takonoasi.sasimi@gmail.com (R.H.); saki_kirakira15@yahoo.co.jp (S.S.); wagatuma@tds1.tr.yamagata-u.ac.jp (T.W.); oikawa@tds1.tr.yamagata-u.ac.jp (A.O.); 2 RIKEN Center for Sustainable Resource Science, Yokohama 230-0045, Japan; E-Mail: kazuki.saito@riken.jp

**Keywords:** *Phaseolus vulgaris*, phosphorus deficiency, rhizosphere, root exudates

## Abstract

Root exudates improve the nutrient acquisition of plants and affect rhizosphere microbial communities. The plant nutrient status affects the composition of root exudates. The purpose of this study was to examine common bean (*Phaseolus vulgaris* L.) root exudates under phosphorus (P) deficiency using a metabolite profiling technique. Common bean plants were grown in a culture solution at P concentrations of 0 (P0), 1 (P1) and 8 (P8) mg P L^−1^ for 1, 10 and 20 days after transplanting (DAT). Root exudates were collected, and their metabolites were determined by capillary electrophoresis time-of-flight mass spectrometry (CE-TOF MS). The shoot P concentration and dry weight of common bean plants grown at P0 were lower than those grown at P8. One hundred and fifty-nine, 203 and 212 metabolites were identified in the root exudates, and 16% (26/159), 13% (26/203) and 9% (20/212) of metabolites showed a P0/P8 ratio higher than 2.0 at 1, 10 and 20 DAT, respectively. The relative peak areas of several metabolites, including organic acids and amino acids, in root exudates were higher at P0 than at P8. These results suggest that more than 10% of primary and secondary metabolites are induced to exude from roots of common bean by P deficiency.

## 1. Introduction

Phosphorus (P) is an essential macronutrient for plant growth. Phosphate fertilizer is applied to agricultural soils to replenish phosphorus taken up by plants through their roots. Phosphate fertilizer is produced from phosphate rock. Global P production is predicted to peak around 2030 [1]. It is necessary to plan agricultural strategies in response to the exhaustion of phosphate rock in the future. Firstly, the rate of P fertilizer application should be reduced; secondly, the low-P tolerance of plants should be improved; and finally, a system of recycling P resources must be established. The low-P tolerance of plants is mainly determined by the ability of plants to acquire P from soil and use P efficiently [2].

Root morphological changes, such as root elongation, branching and root hair formation, and physiological changes, such as increased expression levels of the P influx transporter in roots, release of organic acids and phosphatase, which can change soil P from its unavailable form to its available form, and association with mycorrhizal fungi are the mechanisms by which plants acquire P from soil. Remodeling of P, accumulation of starch and the alternative pathways of carbon metabolism are the mechanisms underlying efficient P use. Organic acids and phosphatase are the P acquisition metabolites among various kinds of metabolites in root exudates. The roles of other metabolites in low-P tolerance are not yet clarified. It is necessary to detect the changes of metabolites in root exudates under P deficiency. Stress-related metabolites, such as polyols and sugars, are accumulated in P-deficient roots of common bean [3]. Common bean plants grown under a high N supply increased total sugar and organic acid exudation [4]. Fumaric acid [5], oxalic acid and maleic acid [6] were detected in root exudate of common bean. However, root exudation of common bean under P-deficient conditions is still not known.

Metabolomics is the unbiased identification and quantification of all metabolites in a biological material. The capillary electrophoresis/time-of-flight mass spectrometry (CE-TOF MS) is a useful analytical method of separating and detecting a wide range of ionic metabolites, such as amino acids, organic acids, sugar phosphates and nucleotides [7]. CE-TOF MS has been used for various plant studies: the clarification of metabolite composition in rice leaves [8] and elucidation of the localization and dynamics of metabolites in a single cell of the alga, *Chara australis* [9]. CE-TOF MS is also useful to determine metabolites in root exudates [10].

The common bean (*Phaseolus vulgaris* L.) is the most important leguminous crop for human consumption in the world. The common bean comprises 50% of the grain legumes consumed worldwide [11]. The low-P tolerance of the common bean is lower than that of other crop species [12]. Low P availability in soil is often a major constraint of its growth [13]. The purpose of the present study is to apply metabolite profiling using CE-TOF MS to the analysis of root exudates and to clarify the composition of common bean root exudates under P deficiency.

## 2. Results and Discussion

### 2.1. Shoot Growth and P Status of Common Bean

Seven-day-old common bean plants were grown in nutrient solution at 0 (P0), 1 (P1) and 8 (P8) mg P L^−1^ for 1, 10 and 20 days. Shoot dry weight increased at all P concentrations 20 days after transplanting (DAT) compared with that for 1 DAT (Table 1). Shoot dry weight was not significantly different among plants grown at P0, P1 and P8 1 DAT. The shoot dry weight was lower in plants at P0 than in those at P8 10 and 20 DAT. The shoot P concentration decreased at all P concentrations 20 DAT compared with that for 1 DAT. The shoot P concentration was lower in plants at P0 than in those at P8 1, 10, and 20 DAT. The shoot P concentration was 4.75 mg P g^−1^ in plants at P0 1 DAT and dropped to 1.83 mg P g^−1^ 10 DAT (Table 1). A shoot P concentration lower than 2.5 mg P g^−1^ in the common bean is considered to be deficient [14]. The shoot P concentration in plants at P0 1 DAT was not lower than 2.5 mg P g^−1^, but lower than that in plants at P8. The effect of the P concentration of the nutrient solution on the P status of plants appeared only one day after treatment, and the concentration increased until 20 DAT.

**Table 1:** Shoot dry weights and shoot P concentrations of common bean plants grown in culture solution at three P concentrations, 1, 10 and 20 days after transplanting (DAT). The means ± standard error of six biological replicates are shown. Means followed by the same letter are not significantly different (*p* < 0.05) by the Tukey test.

DAT	P concentration	Shoot dry weight				Shoot P concentration			
	(mg P L^-1^)	(mg/plant)				(mg P/g)			
1	0	0.208	±	0.008	a	4.75	±	0.28	b
1	1	0.194	±	0.019	a	5.58	±	0.31	b
1	8	0.202	±	0.013	a	6.50	±	0.06	a
10	0	0.480	±	0.019	b	1.83	±	0.13	c
10	1	0.558	±	0.048	b	2.69	±	0.22	b
10	8	0.919	±	0.067	a	6.33	±	0.25	a
20	0	0.861	±	0.111	b	1.35	±	0.04	b
20	1	0.840	±	0.006	b	1.86	±	0.2	b
20	8	1.943	±	0.176	a	2.85	±	0.39	a

### 2.2. Response of Metabolites in Root Exudates to P Deficiency

One hundred and fifty-nine, two hundred and three and two hundred and twelve metabolites were detected in root exudates of the common bean 1, 10 and 20 DAT, respectively (Tables S1, S2 and S3). The relative peak areas of metabolites increase, decrease or did not change in response to the P concentration of the nutrient solution. The P0/P8 response ratio of the relative peak area was calculated. Twenty-six (16% of total) metabolites showed a ratio higher than 2.0 1 DAT (Table 2 and Table S1), indicating an increase in the ratio in P-deficient plants, whereas 20 (13%) metabolites showed a ratio lower than 0.5. No 2-aminobutyrate, 3-hydroxypropionate, 3-methyladenine, cadaverine, castanospermine, NAD, spermidine or trans-zeatin was detected in plants grown at P8. The relationship of these metabolites with the P status of plants is not known. On the other hand, no cystine, fructose-1,6-biphosphate (F1,6P) + glucose-1,6-biphosphate (G1,6P), *N*-acetyl-D-glucosamine 6-phosphate (GlcNAc6P), phenylphosphate (phenylP) or pimelate was detected in plants grown at P0. The relative peak areas of 113 (71%) metabolites did not change regardless of the P concentration of the nutrient solution.

**Table 2:** Fold change (0 (P0) mg P L^−1^/P8) of the relative peak area of metabolites detected in root exudates of common bean 1, 10 and 20 days after transplanting. Metabolites that have a fold change >2.0 (pink) and < 0.5 (blue) are shown.

Metabolite	1	10	20	Metabolite	1	10	20	Metabolite	1	10	20
Amino acid, amine, amino and acid derivative				Oxamate	1.88	0.82	2.09	2-Aminoadipate	0.46	0.48	0.51
Cadaverine	20.00	0.00	0.79	Pipecolate	0.44	0.33	0.43	2-Aminobutyrate	20.00	0.00	0.20
Cyclohexylamine	1.53			Shikimate		0.27	20.00	3-Hydroxypropionate	20.00	0.25	0.31
Diethanolamine	1.16	0.82	0.40	Sinapate		20.00	1.21	3-Methyladenine	20.00	0.17	0.08
Isobutylamine	3.60		10.00	Succinate	0.58	0.25	0.37	3-MethylHis		1.12	0.46
Serotonin		3.43	1.09	Tartrate	2.69	1.15	2.27	4-Amino-3-hydroxybutanoate	2.59	0.55	0.60
Allantoate		1.51	4.39	trans-Cinnamate		0.83	0.50	4-Coumarate;Coumarate		20.00	1.16
Arginine	1.03	3.45	4.85	P compound				4-Hydroxybenzoate	1.57	20.00	0.49
Asparagine	1.53	6.65	6.59	ADP＋dGDP	0.99	0.36	0.46	5-Amino-4-oxovalerate		0.20	0.37
Citrulline	1.67	2.33	1.88	AMP	0.93	0.00	0.56	5-Aminopentanoate	5.28	0.03	1.27
Creatine	1.92	0.60	0.16	cCMP	0.90		0.00	6-Hydroxynicotinate		1.00	20.00
Creatinine	2.01	0.80	0.47	cGMP	2.78			ABA	0.75	7.79	12.02
Cysteine		1.11	0.00	CMP	0.52	0.22	0.42	Acetoacetate;2-Oxobutyrate	1.19	0.00	20.00
Cystine	0.00	0.84	0.33	F1,6P;G1,6P	0.00	0.00	0.00	Allantoin	1.16	2.20	3.44
DimethylGly	1.94	0.32	0.22	G1P＋Gal1P	0.37	0.00	0.19	Anthranilate＋Trigonelline	0.80	0.61	0.35
GABA	1.58	0.68	0.47	G6P＋F6P＋M6P	0.25	0.00	0.00	Atropine		0.77	0.45
Glutamine	1.82	3.46	1.17	Galacturonate1P	1.84	0.00	0.66	b-Imidazolelactate		2.23	0.09
Gly-Leu	0.63	1.01	3.21	GlcN6P	1.00	0.35	0.47	Biotin			0.39
Glycine	1.36	0.67	0.31	GlcNAc6P	0.00	0.53	0.17	Carnitine	2.70	0.41	0.65
Histidine	1.97	3.31	0.90	Gly3P	0.70	0.02	0.38	Castanospermine	20.00	1.57	0.44
Ile＋Leu	1.06	0.45	0.32	GMP	0.56	0.50	0.68	CysSG		0.00	0.81
Lysine	1.35	0.61	0.40	IMP	0.47	0.62	1.14	GalN;GlcN	2.97	1.04	0.02
Methionine		0.20	0.06	PhenylP	0.00			Gentisate	1.32	4.32	1.29
O-AcetylSer	0.67	4.15	1.31	Pyridoxamine5P		0.31	0.00	GlcNAc	1.11	0.45	0.47
Ornithine	1.77	1.43	2.38	UDP-Glc;UDP-Gal	1.44	0.45	0.63	Glucosaminate	0.87	20.00	1.69
Phenylalanine	0.97	0.36	0.24	UMP	0.58	0.17	0.35	Glycerate	1.11	0.48	0.44
Proline	1.12	0.12	0.20	Nucleic acid, nucleotide				Harman		0.46	0.18
Threonin	1.18	0.70	0.40	Deoxyadenosine	3.32	2.78	4.44	HMG		0.55	0.37
Tryptophan	0.64	0.50	0.26	Deoxycytidine	1.47	2.49	3.57	Homogentisate			20.00
Tyramine			20.00	Deoxyguanosine	1.98	5.17	4.07	Imidazole-4-acetate	2.15	1.00	0.46
Tyreonine	0.86	0.37	0.25	Guanine	1.00	0.51	0.16	Methionine sulfoxide	0.14	0.33	0.19
Valine	1.05	0.69	0.37	Hypoxanthine	0.79	0.37	0.20	Mugineate		3.03	1.11
N-FormylMet		1.64	10.00	Methylthioadenosine	0.48	0.99	1.05	N-AcetylLeu	2.98	0.36	0.45
Octopine	0.31	0.65	0.29	NAD	20.00			N6-Methyl-2'-deoxyadenosine	0.57	1.04	0.12
Histidinol		20.00	1.08	Homologue				Nicotinamide	0.46	0.11	0.25
Carboxylic acid				HomoGln＋Ala-Ala	0.79	0.85	0.35	Octylamine	3.80	0.00	2.21
2-Furoate	0.69	0.48	1.63	HomoIle;HomoLeu	1.19	1.28	0.27	Ophthalmate		1.34	0.49
2-Hydroxyisobutyrate	1.07	1.49	2.14	HomoLys	0.00	1.40	0.80	PhenylacetylGly	0.48	0.50	0.38
3-Dehydroshikimate	1.08	0.14	0.69	HomoPhe		0.22	0.19	Pimelate	0.00	0.81	0.43
4-Oxovalerate	0.34	0.34	0.18	HomoPro		0.00	1.02	Putrescine	1.18	0.81	0.40
4-Pyridoxate		0.63	0.39	HomoThe	2.77	1.29	0.67	Pyrrolidine		0.25	0.95
Adipate	0.59	0.33	0.48	Vitamine				S-SulfoCys		0.00	0.00
Arginosuccinate			5.47	Choline	0.44	0.51	0.35	SAH		0.49	0.59
Benzoate	0.90	0.43	0.20	Nicotinate	1.16	0.23	0.44	Spermidine	20.00	1.06	0.32
Citrate	2.85	20.00	3.00	Folate		0.00		Stachydrine	0.79	0.99	0.34
Cysteate		0.72	0.37	Sugars				SulfinoAla		1.09	0.42
Fumarate	1.82	0.43	0.66	N-AcetylGlu		0.39	0.87	Trimethylamine-N-oxide	2.48	3.76	4.25
Glutarate	0.18	0.29	0.57	Peptide				tZ	20.00	1.34	0.45
Glyoxylate	2.32	0.33	0.79	Carnosine		1.51	0.33	tZR	1.08	1.19	0.27
Hippurate	1.28	2.07	20.00	Others				Urocanate	1.09	0.41	0.72
Itaconate	1.35	1.85	2.45	Agmatine		4.61	1.15	Vanillate			20.00
Lactate	1.72	20.00	0.64	IsoAsn		0.84	0.42	Vanillylmandelate			20.00
Malonate	1.60	0.58	0.37	O-SuccinylhomoSer	0.27	0.90	0.33	Xanthine		0.00	0.44
Mevalonolactone	2.53	0.54	0.15	1-Aminopropanediol		20.00	0.97	Xanthurenate	1.22	0.78	0.43
Mucate	1.29	5.48	2.40								

Twenty-six metabolites showed a P0/P8 ratio higher than 2.0, and 60 metabolites showed a P0/P8 ratio lower than 0.5 10 DAT (Table 2 and Table S2). No 1-aminopropanediol, 4-coumarate, 4-hydroxybenzoate, citrate, glucosaminate, histidinol, lactate or sinapate was detected in plants grown at P8. No 2-aminobutyrate, acetoacetate, AMP, cadaverine, CysSG, F1,6P+G1,6P, folate, glucose-1-phosphate (G1P) + galactose-1-phosphate (Gal1P), glucose-6-phosphate (G6P) + fructose-6-phosphate (F6P) + mannose-6-phosphate (M6P), galacturonate-1-phosphate (galacturonate1P), homoproline (homoPro), S-sulfocysteine (S-sulfoCys) or xanthine was detected in plants grown at P0. The relative peak areas of 117 (57%) metabolites did not change regardless of the P concentration of the nutrient solution.

Thirty metabolites showed a P0/P8 ratio higher than 2.0, and 82 metabolites showed a P0/P8 ratio lower than 0.5 at 20 DAT (Table 2 and Table S3). Eight amino acid, amine and amino acid derivatives, nine carboxylic acids and three nucleic acid and nucleotide were increased at P0. Eighteen amino acid, amine and amino acid derivatives, ten carboxylic acids, 11 P compounds and two nucleic acid and nucleotide were decreased at P0. The relative peak areas of 100 (47%) metabolites did not change regardless of the P concentration of the nutrient solution.

**Figure 1:**
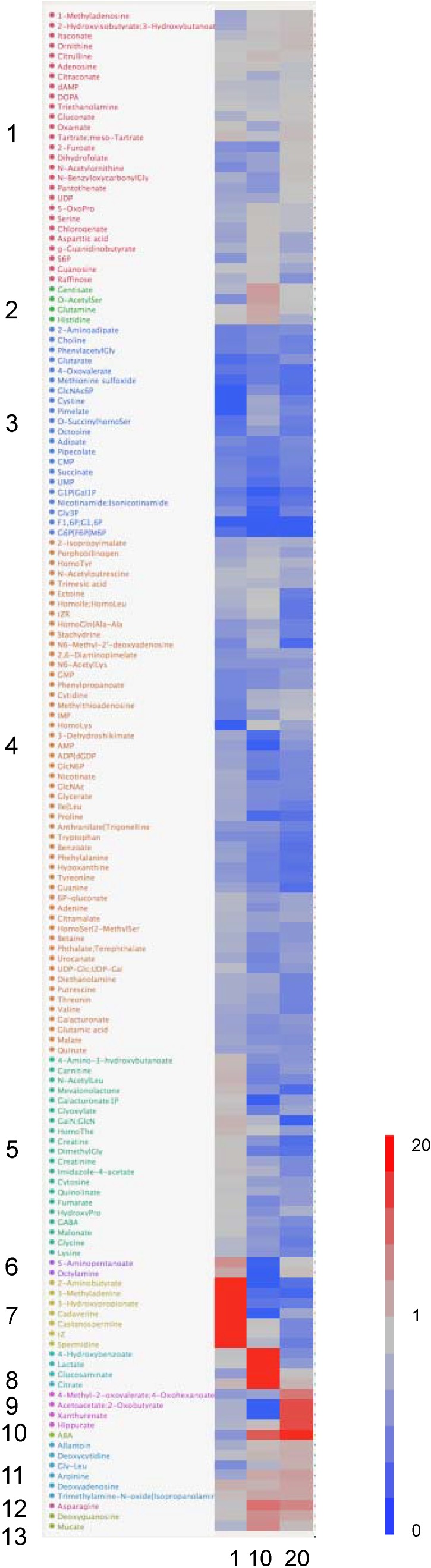
Clustered heat map of the P0/P8 ratios of metabolite relative peak areas for root exudates of common bean plants 1, 10 and 20 DAT. The P0/P8 ratio increased by P deficiency is shown in red and that decreased by P deficiency in blue.

Changes of metabolites in root exudates to the P concentration of plants were classified by hierarchical clustering analysis (HCA) (Figure 1). The P0/P8 ratio of deoxyadenosine, trimethylamine-*N*-oxide + isopropanolamine in Cluster 11 was higher at 1, 10 and 20 DAT. The P0/P8 ratios of ABA, allantoin, deoxycytidine, arginine, deoxyguanosine and mucate in Cluster 9 and Cluster 10 were higher at 10 and 20 DAT. The P0/P8 ratios of 2-aminobutyrate, 3-methyladenine, 3-hydroxypropionate, cadaverine, castanospermine, trans-zeatin and spermidine in Cluster 7 were higher only at 1 DAT. The P0/P8 ratios of 4-hydroxybenzoate, lactate and glucosaminate were higher only at 10 DAT. The P0/P8 ratios of 4-oxovalerate, ADP + dGDP, F1, 6P + G1,6P, G1P + Gal1P, G6P + F6P + M6P, phenylacetylglycine (phenylacetylGly) and pipecolate in Cluster 3 were lower at 1, 10 and 20 DAT. F1, 6P + G1, 6P, G1P + Gal1P and G6P + F6P + M6P are phosphate esters. The concentrations of F6P and pipecolate decreased in P-deficient nodules of common bean plants [15]. The synthesis of these metabolites in the shoots or roots of common bean plants might be inhibited under P deficiency. The anion channels that allow the efflux of nitrate, chloride and malic acid have been identified [16], but their efflux mechanism has still not been identified. Aluminum-activated malate efflux protein was identified from *Triticum aestivum* [17]. Organic acid efflux protein activated by low P might be involved in the root exudation.

Stress-related metabolites, such as polyols and sugars, are accumulated in the P-deficient roots of common bean [3]. However, root exudation of these metabolites under P-deficient conditions and its roles are still not known. P0/P8 ratios of citrate, deoxyadenosine and trimethylamine-N-oxide + isopropanolamine asparagine, deoxycytidine, deoxyguanosine, hippurate, mucate and riboflavin were higher than 2.0 at 10 and 20 DAT. Citrate, malate, malonate and oxalate are usually the organic acids most commonly exuded by plants from roots under P deficiency in order to acquire insoluble inorganic phosphate in soil [18]. The P0/P8 ratios of citrate in common bean root exudates were higher at 1, 10 and 20 DAT, but the relative areas of malate and malonate did not change under P deficiency. Other organic acids, such as hippurate and mucate were also exuded under P deficiency. However, little work has been carried out on the root exudation of these organic acids in response to P deficiency. Amino acids detected in root exudates affect the growth of soil microorganisms. Asparagine increases the respiration rate and induces changes in the bacterial community [19]. The addition of arginine to soil changes the soil community structure of bacteria and fungi [20]. ABA was detected in the root exudates of rice under water stress [21]. Enhanced root exudation of deoxyadenosine, trimethylamine-*N*-oxide + isopropanolamine, ABA, allantoate, allantoin, arginine, asparagine, deoxycytidine, deoxyguanosine, hippurate, mucate and riboflavin in P-deficient plants was shown in this study for the first time. The high L-asparagine amendments resulted in both increased respiration rates and clear changes in both the 16S rRNA and rDNA community fingerprints [19]. The iron deficiency of *Beta vulgaris* increased the root exudation of riboflavin. Riboflavin may act in iron acquisition, but its role in P acquisition is not known.

### 2.3. Functional Roles of Metabolites in Rhizosphere under P Deficiency

We previously reported the metabolite profiling of root exudates of rice plants under P deficiency [10]. P deficiency enhanced root exudation of only asparagine, deoxyadenosine, shikimate, spermidine and tyramine in both rice and common bean plants. There might be a difference in the mechanisms of root exudation under P deficiency between rice and common bean plants. Sixty-five metabolites were identified in the root exudates of rice, and the concentrations of 26 metabolites (40%) were higher in rice plants grown at P0. On the other hand, 16% (26/159), 13% (26/203) and 9% (20/212) of metabolites showed a P0/P8 ratio higher than 2.0 in common bean root exudates at 1, 10 and 20 DAT, respectively. The difference in root exudation under P deficiency between rice and common bean might contribute to the difference of the low P tolerance of these plants.

## 3. Experimental Section

### 3.1. Plant Materials and Growth Conditions

Common bean (*P. vulgaris* L. cv. Yukitebou) seeds were sown in a vermiculite spread and irrigated with deionized water. Three seven-day-old seedlings were transplanted to a 2-L Wagner pot filled with a culture solution. The culture solution developed by Wagatsuma *et al.* [22] contained the following mineral nutrients (mg per liter): 40 N (NH_4_NO_3_), 20 N (NaNO_3_), 60 K (K_2_SO_4_), 80 Ca (CaCl_2_), 40 Mg (MgSO_4_), 2 Fe (FeSO_4_), 1 Mn (MnSO_4_), 0.01 Cu (CuSO_4_), 0.005 Mo ((NH_4_)_6_Mo_7_O_24_), 0.4 B (H_3_BO_3_) and 0.2 Zn (ZnCl_2_). The P concentration of the culture solution was adjusted to 0 (P0), 1 (P1) or 8 (P8) mg of P per liter using NaH_2_PO_4_. The pH of the solution was adjusted daily to 5.0 with 0.1 M NaOH and 0.1 M H_2_SO_4_. The solution was aerated continuously with vinyl chloride tubes connected to an air pump and replaced weekly. Each P treatment had six replicates. Plants were grown in a glasshouse at Yamagata University (38°44'N, 139°50'E) from October 14, 2010, to November 23, 2010 (20 DAT).

### 3.2. Root Exudate Collection

Root exudates were collected at 1, 10 and 20 DAT. Common bean plants were rinsed with sterile deionized water several times. One plant was bound to urethane foam and placed on top of a 100-mL paper cup containing 100 mL of sterile deionized water. The cups were aerated and placed in a dark room for 12 h. The root exudate solution was filtered through filter paper (No. 6 Advantec Toyo, Tokyo, Japan). Fifty milliliters of the solution were transferred to a polyethylene tube and stored at −20 °C. Frozen root exudates were lyophilized.

After collecting the root exudates, the shoots and roots were washed with deionized water and separated with a pair of forceps. Subsamples of the shoots and roots were prepared, and their fresh weights were determined. One-half of the subsamples were frozen immediately at −20 °C, and the other half were dried at 70 °C for 3 days. The frozen shoots and roots were used for the determination of metabolites. The dried shoots were used for the determination of dry weight and P concentration. The dry weight of the shoot subsamples was determined. Ground shoot subsamples were digested with HNO_3_-HClO_4_-H_2_SO_4_ (5:2:1) solution. The P concentration in the digested solution was determined colorimetrically with the vanadomolybdate-yellow assay [23].

### 3.3. Capillary Electrophoresis Mass Spectrometry

Six hundred microliters of methanol containing 8 µM methionine sulfone as the internal standard, 600 µL of chloroform and 200 µL of Milli-Q water were added to the freeze-dried root exudates. The upper layer of each solution was transferred to a 1.5-mL test tube, evaporated for 30 min with a centrifugal concentrator and then separated into two layers. The upper layer was centrifugally filtered through a Millipore 5-kD cutoff filter at 9100× *g* at 4 °C. The filtrate was dried with the centrifugal concentrator. The residue was dissolved in 20 µL of Milli-Q water containing a reference compound (3-aminopyrrolidine). The CE-MS system and its conditions were as described by Watanabe *et al.* [24] and Oikawa *et al.* [9].

All CE-TOF MS experiments were performed using an Agilent capillary electrophoresis system (Agilent Technologies, Waldbronn, Germany), an Agilent G3250AA LC/MSD TOF system, an Agilent 1100 series binary HPLC pump, a G1603A Agilent CE-MS adapter and a G1607A Agilent CE-ESI-MS sprayer kit. The G2201AA Agilent ChemStation software for CE and the Analyst QS software for TOF MS were used. Separations were carried out using a fused silica capillary (50 mm i.d. × 100 cm total length) filled with 1 M formic acid for cation analyses or with 20 mM ammonium formate (pH 10.0) for anion analyses as the electrolyte. The sample solutions were injected at 50 mbar for 15 s (15 nL). Prior to each run, the capillary was flushed with the electrolyte for 5 min. The applied voltage was set at 30 kV. The capillary temperature was maintained at 20 °C, and the sample tray was cooled to below 4 °C. Fifty percent (*v*/*v*) methanol/water containing 0.5 mM reserpine was delivered as the sheath liquid at 10 mL/min. ESI-TOF MS was conducted in the positive ion mode for cation analyses or in the negative ion mode for anion analyses, and the capillary voltage was set at 4 kV. The flow rate of the heated dry nitrogen gas (heater temperature, 300 °C) was maintained at 10 psig. In TOF MS, the fragmentor, skimmer and Oct RFV voltages were set at 110 V, 50 V and 160 V for cation analyses or at 120 V, 60 V and 220 V for anion analyses, respectively. Each acquired spectrum was automatically recalibrated using the reference masses of reference standards. The methanol dimer ion ([2M + H]^+^, *m*/*z* = 65.0597) and reserpine ([M + H]^+^, *m*/*z* = 609.2806) for cation analyses or the formic acid dimer ion ([2M − H]^−^, *m*/*z* = 91.0037) and reserpine ([M − H]^−^, *m*/*z* = 607.2661) for anion analyses provided the lock mass for exact mass measurements. Exact mass data were acquired at a rate of 1.5 cycles/s in the range of 50–1,000 *m*/*z*. Metabolites were identified and quantified as described by Oikawa *et al.* [9].

## 4. Conclusions

A high-resolution metabolite profiling has been performed to clarify the effect of P nutrition on the changes of metabolites in common bean root exudates. The non-target CE-MS method was employed to separate and detect ionic metabolites, including amino acids, organic acids, nucleotides and sugar phosphates, without any treatments for chemical derivatization.

Common bean root exudates were collected from P-sufficient and P-deficient common bean plants with solution culture. More than 150 metabolites were detected in root exudates. The responses of metabolites in root exudates to the P deficiency of plants differed among metabolites and among growth periods. The relative peak areas of several metabolites, including organic acids and amino acids, in root exudates were higher at P0 than at P8. These results suggest that more than 10% of primary and secondary metabolites are induced to exude from the roots of common bean by P deficiency.

## Supplementary Files

Supplementary File 1
